# Depression, violence and cortisol awakening response: a 3-year longitudinal study in adolescents

**DOI:** 10.1017/S0033291718001654

**Published:** 2018-07-17

**Authors:** Rongqin Yu, Susan Branje, Wim Meeus, Philip Cowen, Seena Fazel

**Affiliations:** 1 Department of Psychiatry, University of Oxford, Oxford, UK; 2 Department of Youth and Family, Utrecht University, Utrecht, The Netherlands; 3 Department of Developmental Psychology, Tilburg University, Tilburg, The Netherlands

**Keywords:** Adolescence, aggression, cortisol awakening response, depression, violence

## Abstract

**Background:**

Despite evidence of links between depression and violent outcomes, potential moderators of this association remain unknown. The current study tested whether a biological marker, cortisol, moderated this association in a longitudinal sample of adolescents.

**Methods:**

Participants were 358 Dutch adolescents (205 boys) with a mean age of 15 years at the first measurement. Depressive symptoms, the cortisol awakening response (CAR) and violent outcomes were measured annually across 3 years. The CAR was assessed by two measures: waking cortisol activity (CAR area under the curve ground) and waking cortisol reactivity (CAR area under the curve increase). Within-individual regression models were adopted to test the interaction effects between depressive symptoms and CAR on violent outcomes, which accounted for all time-invariant factors such as genetic factors and early environments. We additionally adjusted for time-varying factors including alcohol drinking, substance use and stressful life events.

**Results:**

In this community sample, 24% of adolescents perpetrated violent behaviours over 3 years. We found that CAR moderated the effects of depressive symptoms on adolescent violent outcomes (*β*s ranged from −0.12 to −0.28). In particular, when the CAR was low, depressive symptoms were positively associated with violent outcomes in within-individual models, whereas the associations were reversed when the CAR was high.

**Conclusions:**

Our findings suggest that the CAR should be investigated further as a potential biological marker for violence in adolescents with high levels of depressive symptoms.

## Introduction

Adolescent depression has been linked to a wide range of negative outcomes including suicide (Hawton *et al.*, 2012), substance use (Henry *et al.*, 1993) and various social impairments (Rudolph, 2009). Increasing evidence suggests that depression is also associated with an elevated risk of violent outcomes. The increased risk of violence has been reported in several longitudinal studies (Capaldi and Stoolmiller, 1999; Kofler *et al.*, 2011; Fazel *et al.*, 2015; Yu *et al.*, 2017) including using sibling and twin designs (Fazel *et al.*, 2015). In addition, other designs, including cross-sectional and prevalence studies in selected samples, are consistent with this link (Arseneault *et al.*, 2000; Coid *et al.*, 2006; Fairchild *et al.*, 2008; Ferguson *et al.*, 2009; Piko and Pinczés, 2014). However, inconsistent findings have been reported, as several studies found no clear associations between depression and violent outcomes (Chen *et al.*, 2012; van Dorn *et al.*, 2012; Marsh *et al.*, 2016). Clarifying moderators of the links between depression and violent outcomes is needed and may assist in the design of effective prevention and intervention programmes.

One potentially important moderator of the link between depression and violent outcomes could be differences in stress sensitivity (Raine, 2002; Hellhammer *et al.*, 2009). Blunted hypothalamic–pituitary–adrenal (HPA) activity is a component of stress hypoarousal that may reduce the ability to share the distress of others (von Polier *et al.*, 2013; Johnson *et al.*, 2014). Cortisol is the primary hormonal end product of the HPA axis and its response or activity is an important biological indicator of self-regulation, playing a central role in the regulation of emotional and behavioural responses to environmental stressors. Low cortisol activity is an indication of blunted HPA activity and is hypothesized to be linked to antisocial behaviours (Raine, 2002). The cortisol awakening response (CAR) refers to the marked morning rhythm normally exhibited by cortisol within the first hour after awakening, and is characterized by a rapid increase in levels upon awakening, peaking at around 30 min post-awakening, and declining thereafter (Wüst *et al.*, 2000). The overall volume of cortisol released over the waking period [area under the curve ground (AUCg)] and the absolute changes in cortisol levels post-awakening [area under the curve increase (AUCi)] provide useful and reliable markers of HPA activity (Pruessner *et al.*, 1997).

Support for the association between CAR and antisocial behaviours comes from studies that report lower morning cortisol levels and increased risk of violent outcomes in clinical samples with attention-deficit hyperactivity and conduct disorders (Pajer *et al.*, 2001; Freitag *et al.*, 2009) and general community samples (Shirtcliff *et al.*, 2005; Platje *et al.*, 2013). In particular, several longitudinal studies have reported that low morning cortisol levels predicted adolescent aggressive behaviours 5 years later (Shoal *et al.*, 2003) and persistent aggressive behaviours over 3 years (Platje *et al.*, 2013). However, this is not a consistent finding. For instance, studies in predominantly male samples of adolescent offenders and disruptive youth have shown non-significant links (Dabbs *et al.*, 1991; Scerbo and Kolko, 1994).

Previous studies have shown that psychological factors such as depression and biological features such as CAR can independently lead to aggressive and violent behaviours. From a psychobiological point of view, aggressive and violent behaviours can also be regarded as the outcome of the interaction between psychological and biological factors (Quay, 1993). Thus, depression is likely to interact with CAR in predicting adolescent violent outcomes. Based on prior research (Platje *et al.*, 2013; Fazel *et al.*, 2015), we propose the following hypotheses on the patterns of interaction. Specifically, when adolescents have higher levels of depressive symptoms, they have an increased risk of violent outcomes, in particular when their CAR decreases. The theoretical basis for this is that as depressive symptoms include pervasive low mood, negativity, irritability, agitation and pessimism, when these symptoms are experienced in combination with low CAR, adolescents will have more difficulty dealing with or regulating these symptoms and be more likely to act out their distress with violence and aggression towards others. In contrast, high CAR may act as a protective factor in the link between depressive symptoms and violence. In particular, it might serve as a biological buffer to prevent individuals from acting out depressive symptoms with violent behaviours. Thus, we hypothesize that depression is not associated with violent outcomes when CAR is higher. We tested these hypotheses with the data from a longitudinal study of adolescents. Understanding the interplay between cortisol response and depressive symptoms is important as it could contribute to the possibility of using biomarkers as part of prognostic assessments in mental health (Vitacco *et al.*, 2015).

## Methods

### Sample

Participants were 358 adolescents (205 boys) who took part in cortisol awaking measurements at wave 3 of the ongoing longitudinal RADAR Young study (*N* = 497, Research on Adolescent Development And Relationships). RADAR Young is a cohort study focusing on adolescent developmental outcomes including internalizing and externalizing problem behaviours and physiological developments. The current study was based on the data from the third to fifth annual waves. The mean age of the participants in wave 3 was 15.0 years (ranging from 14.0 to 17.6; s.d. = 0.5). All participants identified themselves as Dutch. In this sample, 10.5% were from low socioeconomic status family, which was defined as having a father and mother who were unemployed or held a manual job (Statistics Netherlands, 1993). In addition, analyses of all variables used in this study revealed a normed χ^2^ (χ^2^/df) of 1.04, which indicates that the pattern of the missing data was not materially different from a missing completely at random pattern (Bollen, 1989).

### Procedure

Participants were recruited from various Dutch elementary schools. Across 3 years at a similar time of each year, adolescents filled out questionnaires on socio-demographic, depression and violence measures during annual home visits, supervised by trained research assistants. In addition to the administration of the behavioural measurements, research assistants gave detailed verbal and written instructions for cortisol measurements. The RADAR study has been approved by the medical ethics committee of the University Medical Center in Utrecht, The Netherlands.

### Measurements

#### Depressive symptoms

The Reynolds Adolescent Depression Scale, second edition (RADS-2) (Reynolds, 2002) was used to measure depression symptoms. This self-report questionnaire includes 23 items (e.g. ‘I feel nobody cares about me.’) Adolescents responded to the questionnaire on a four-point Likert scale, ranging from 1 (*almost never*) to 4 (*usually*). Scores can range from 23 to 92. Previous research has shown good psychometric properties of RADS-2 (e.g. test–retest reliabilities >0.7 in diverse samples) (Reynolds, 2004). In the current sample, the Cronbach's *α* of this scale was 0.9 in all three annual waves and the average test–retest reliability with a 1-year interval was 0.7 across waves.

#### Violent behaviours

Adolescents’ violent behaviours in the last 12 months were measured on a self-reported scale based on a large international comparative study on delinquency (Enzmann *et al.*, 2010). Violent behaviours were measured with five items including: stealing from person with threat/force, assaulting, injuring someone with a weapon, and beating and/or kicking (with/without) causing injury. Adolescents responded on a five-point scale, ranging from 0 (*never*) to 4 (*more than ten times*). Scores can range from 0 to 20. The Cronbach's *α*s of this scale were 0.5 at wave 3, 0.7 at wave 4 and 0.6 at wave 5 and the average test–retest reliability with a 1-year interval was 0.5 across 3 years.

#### Physical aggression

Physical aggression was measured with a self-reported questionnaire (Linder *et al.*, 2002) via six items (e.g. ‘I push or punch others to get what I want.’) Adolescents responded to these items on a seven-point Likert scale, ranging from 1 (*not at all true*) to 7 (*very true*). Scores can range from 6 to 42. Prior research has indicated good reliability and validity (Linder *et al.*, 2002). The Cronbach's *α* for the scale was 0.9 in all three waves and the average test–retest reliability with a 1-year interval was 0.7 across the three waves.

#### Cortisol awakening responses

CAR_AUCg_ and CAR_AUCi_ were measured in the saliva that was collected by passive drooling, immediately after awakening (Cort0), 30 min (Cort30) and 60 min (Cort60) later. Cortisol sampling took place in February and March of each consecutive year, as soon as possible after assessing depression and violent outcomes from wave 3 to 5. The saliva sampling was scheduled on a typical weekday during the school year. Participants were instructed to rinse their mouths with water before sampling, and not to eat, drink, smoke or brush their teeth before completing Cort60. They were requested to collect their saliva through a small straw into a polypropylene tube, and label these tubes with the time and date of sampling. After collection, participants were asked to store the samples in the refrigerator and send them by mail to the research centre the same day. At the research centre, the cortisol collections were stored uncentrifuged at −20 °C until analysis. Salivary cortisol levels were analysed using electrochemiluminescence immunoassay (E170 Roche, Switzerland). The lower detection limit was 0.5 nmol/l, and the mean intra-assay and inter-assay coefficients of variation were 3% and 12%, respectively. Cases were excluded from analyses if the cortisol data used incorrect sampling time, or if it was unclear how it was sampled (i.e. not registered) or contaminated (e.g. by smoking or brushing teeth). In the current study, 358 participants provided qualified data. CAR_AUCg_ is a summary parameter of the repeated measurements of CAR. Thus, it is an estimation of total adrenal cortisol secretion during the first hour after awakening. CAR_AUCi_ is the absolute change in cortisol levels during the first hour post-awakening. We calculated the CAR_AUCg_ and CAR_AUCi_ with the formula provided by Pruessner *et al.* (Pruessner *et al.* 2003). Specifically, CAR_AUCg_ = (Cort30 + Cort0)/2 + (Cort60 + Cort30)/2 and CAR_AUCi_ = (Cort30 + Cort0)/2 + (Cort60 + Cort30)/2 − (3–1) × Cort0.

#### Time dynamic covariates

As alcohol drinking, substance use and stressful life experiences might affect both CAR (Clow *et al.*, 2004; Platje *et al.*, 2013) and violent outcomes (Hoffmann and Cerbone, 1999), the effects of these factors were adjusted. Alcohol use over the last 4 weeks was assessed with a question with six response options, ranging from ‘*none*’ to ‘*daily*’. Substance use was defined as illicit drug use that was assessed with six questions (e.g. How many times have you used XTC/marijuana/cocaine/mushrooms/amphetamine/heroin in the last 12 months?). Responses range from ‘0 *time*’ to ‘40 *times or more*’. Stressful experiences included sexual assault, physical assault and being threatened with violence, and were measured with the International Crime Victims Survey (Nieuwbeerta, 2002). Participants were asked to indicate their stressful life experiences with five items (e.g. Has anyone ever touched you against your will in any sexual way in the past year?). Responses include ‘*yes*’ and ‘*no*’. These time-varying factors were measured at the same three annual waves as the other predictors.

### Statistical analyses

We adopted a within-individual design applying fixed-effects methods to examine the interaction effects between depressive symptoms and CAR on adolescent violent outcomes. Unlike between-individual approaches, estimators in the within-individual model rely only on within-individual changes over time. Adolescence offers a promising period for using this design as individuals pass through significant changes in all the key studied variables (Moffitt, 1993; Shirtcliff *et al.*, 2012; Thapar *et al.*, 2012; Platje *et al.*, 2013).

In addition, in the within-individual model, each individual acts as their own control. As no changes over time occur in time-invariant variables, the effects of them are automatically controlled for in the within-individual design (Gunasekara *et al.*, 2013). Further details of the rationale and regression equations of this method are reported elsewhere (Allison, 2009). As many time-invariant confounding factors, such as genetic and early environmental factors (Risch *et al.*, 2009; Roisman *et al.*, 2009; Byrd and Manuck, 2014; St Clair *et al.*, 2015), might be linked to the studied variables, this method provides parameter estimates that are less subject to bias (Allison, 2009; Gunasekara *et al.*, 2013).

We further extended our model to adjust for time-varying factors including alcohol drinking, hard drug use and stressful life events at each of the three measurement points. In addition, as gender differences have been suggested in the developmental changes of and interactions among studied variables (Dodge *et al.*, 2006; Card *et al.*, 2008; Avenevoli *et al.*, 2015), as well as in other unmeasured time-varying variables such as sex hormones including testosterone and oestradiol (Popma *et al.*, 2007; Mehta *et al.*, 2015; Tackett *et al.*, 2015), we conducted within-individual analyses for boys and girls separately. We also tested three-way interactions between depression, CAR and gender to assess whether the gender differences were statistically significant. Furthermore, we examined whether the interaction effects between depression and CAR differed by age. In addition, as within-individual correlations between CAR_AUCi_ and CAR_AUCg_ were small to moderate (*r* was 0.42 in girls and 0.16 in boys; Table 1), we included CAR_AUCg_ and CAR_AUCi_ in the same model. Data were analysed using STATA SE version 14 (StataCorp, 2015).

**Table 1.** Descriptives of and correlations between depressive symptoms, cortisol awakening responses (CAR), violence behaviours and physical aggression

		Descriptives								Correlations						
		Girls				Boys										
		Mean	SD	Min	Max	Mean	SD	Min	Max	1	2	3	4	5	6	7
1	Depression	39.2	14.2	23	87	32.2	8.4	23	65		**0.10**	−0.02	**0.09**	**0.23**	0.06	**0.12**
2	CAR_AUCg_	1155.3	470.6	200.6	3100.5	1038.0	391.6	1044.0	2540.4	−0.06		**0.16**	0.06	0.05	0.07	0.09
3	CAR_AUCi_	13.7	455.0	−2096.4	1637.6	−101.7	377.9	−2277.3	752.6	−0.02	**0.42**		**0.09**	0.07	**0.10**	0.05
4	Violence	0.2	0.9	0	8	0.7	1.8	0	20	**0.31**	−0.01	−0.09		**0.42**	**0.21**	**0.30**
5	Aggression	7.6	3.6	6	27	10.2	5.6	6	39	**0.27**	**0.11**	0.06	**0.38**		**0.16**	0.08
6	Alcohol drinking	0.9	0.8	0	4	1.2	0.9	0	5	−0.01	0.**18**	**0.17**	0.03	**0.20**		**0.31**
7	Substance use	1.2	3.5	0	37	1.9	4.6	0	53	**0.17**	0.03	0.08	0.09	**0.18**	**0.27**	
8	Stressful life events	0.1	0.3	0	1	0.2	0.4	0	1							

s.d., standard deviation; CAR_AUCg_, cortisol awakening response area under the curve ground; CAR_AUCi_, cortisol awakening response area under the curve increase. The descriptives and correlations were based on 445 observations in 153 girls and 595 in 205 boys. Data for stressful life events were not included in the correlational matrix due to their binary feature. Coefficients in bold are significant (*p* < 0.05). Correlations regarding girls’ data are presented below the diagonal, regarding boys’ data above the diagonal.

When the interaction effects between depression and CAR were significant, we examined the shape of the interaction by probing the interaction effects. To examine this, simple slopes for each interaction were presented [at 1 standard deviation (s.d.) above and below mean of the moderators (CAR_AUCg_ and CAR_AUCi_)]. Moreover, we applied the Johnson–Neyman technique, using the computational tool of Preacher *et al.* (Preacher *et al.*, 2006) to calculate the values of moderators (CAR_AUCg_ and CAR_AUCi_) at which the regression of outcomes (violence and aggression) on predictors (depressive symptoms) moves from non-significance to significance (http://www.quantpsy.org/interact/mlr2.htm) (Hayes and Matthes, 2009).

## Results

### Descriptive statistics

Table 1 shows an overview of means of and bivariate intercorrelations among depressive symptoms, CAR and violent outcomes for adolescents across three waves. This descriptive information and correlations were based on observations (including repeated measurements of individuals) across three time points. In this community sample, 24% of adolescents perpetrated violent behaviours over 3 years. There were positive correlations between depression and violent outcomes. In general, there was no association between CAR and depression, expect for a small correlation between depression and CAR_AUCg_ in boys (*r* = 0.10) and no association between CAR and violent outcomes, expect for small correlations between CAR_AUCg_ and aggression in girls (*r* = 0.11) and CAR_AUCi_ and violence in boys (*r* = 0.09). Table 2 and Table 3 present the results of our final models examining the interaction effects between CAR and depressive symptoms on adolescent violent outcomes.

**Table 2.** Interaction effects between depressive symptoms and cortisol awakening response (CAR) on adolescent violence

*N* = 358	Violence			
	Girls (*n* = 153)		Boys (*n* = 205)	
	B (s.e.)	*β*	B (s.e.)	*β*
Depression	−0.03 (0.10)	−0.03	0.03 (0.10)	0.02
CAR_AUCg_	−0.01 (0.06)	−0.02	−0.01 (0.08)	−0.01
CAR_AUCi_	**−0.14 (0.05)**	−0.14*	0.08 (0.08)	0.04
Depression × CAR_AUCg_	−0.00 (0.06)	0.00	−0.08 (0.07)	−0.04
Depression × CAR_AUCi_	**−0.26 (0.06)**	−0.28***	0.00 (0.07)	0.00
Alcohol drinking	0.11 (0.06)	0.11	0.09 (0.08)	0.05
Substance use	0.03 (0.09)	0.03	−0.08 (0.12)	−0.04
Stressful life events	**0.61 (0.19)**	0.64**	0.29 (0.22)	0.16

CAR_AUCg_, cortisol awakening response area under the curve ground; CAR_AUCi_, cortisol awakening response area under the curve increase.

***p < 0.001; **p < 0.01; *p < 0.05.

**Table 3.** Interaction effects between depressive symptoms and cortisol awakening response (CAR) on adolescent physical aggression

*N* = 358	Physical aggression			
	Girls (*n* = 153)		Boys (*n* = 205)	
	B (s.e.)	*β*	B (s.e.)	*β*
Depression	−0.10 (0.28)	−0.03	0.55 (0.34)	0.10
CAR_AUCg_	−0.05 (0.16)	−0.01	−0.02 (0.27)	0.00
CAR_AUCi_	**−0.40 (0.15)**	−0.11**	0.10 (0.27)	0.02
Depression × CAR_AUCg_	0.06 (0.16)	0.02	**−0.65 (0.24)**	−0.12**
Depression × CAR_AUCi_	**−0.57 (0.17)**	−0.16**	−0.03 (0.22)	−0.01
Alcohol drinking	0.29 (0.18)	0.08	0.25 (0.27)	0.04
Substance use	**0.72 (0.25)**	0.20**	0.09 (0.40)	0.02
Stressful life events	−0.14 (0.53)	−0.04	0.61 (0.74)	0.11

CAR_AUCg_, cortisol awakening response area under the curve ground; CAR_AUCi_, cortisol awakening response area under the curve increase.

**p < 0.01; *p < 0.05.

### Interaction effects between CAR and depressive symptoms on adolescent violent outcomes

Three of the four models showed significant interaction effects between CAR and depressive symptoms in predicting adolescent violent outcomes at the within-individual level over 3 years, showing that increases in depressive symptoms were associated with elevated risk of violent outcomes when CAR was low. The interaction effects between CAR_AUCi_ and depressive symptoms on violent outcomes including both violence and aggression were significant in girls but not in boys (Table 2). The interaction effect in predicting violence was marginally stronger in girls than boys [B (s.e.) = −0.20 (0.11), *β* = −0.13, *p* = 0.07]. In addition, there was a significant interaction effect between CAR_AUCg_ and depressive symptoms in predicting aggression in boys, whereas no such interaction was present in girls (Table 3). Three-way interaction analyses indicated that this effect was significantly stronger in boys than girls [B (s.e.) = 1.02 (0.36), *β* = 0.20, *p* < 0.01].

**Fig. 1.**
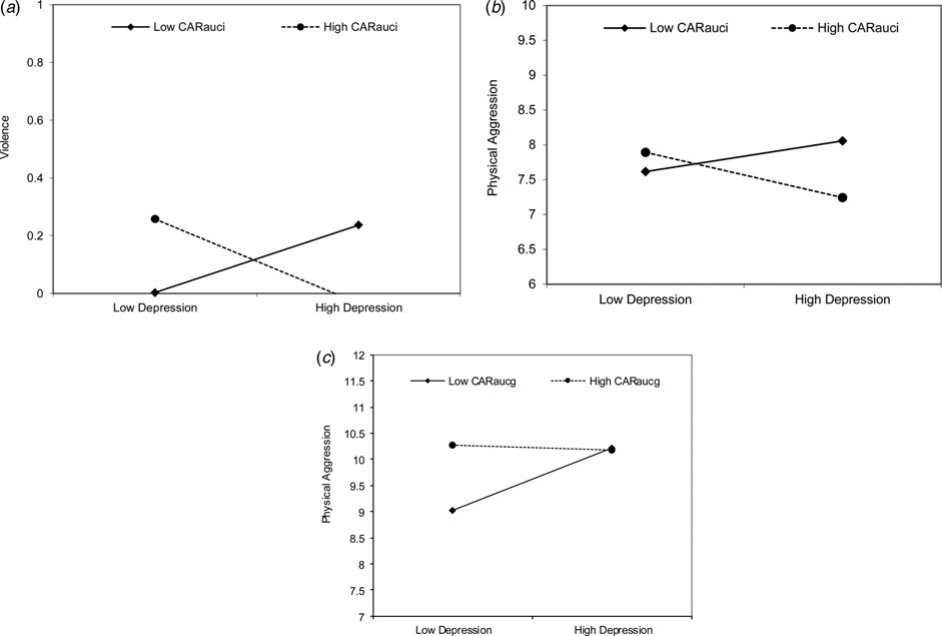
(*a*) Interaction effects between depression and CAR_AUCi_ in predicting violence in girls. (*b*) Interaction effects between depression and CAR_AUCi_ in predicting aggression in girls. (*c*) Interaction effects between depression and CAR_AUCg_ in predicting aggression in boys. (*a*–*c*) CAR_AUCi_, cortisol awakening response area under the curve increase; CAR_AUCg_, cortisol awakening response area under the curve ground. Low, one s.d. below mean; High, one s.d. above mean.

These interaction effects indicated that the associations between depressive symptoms and adolescent violent outcomes differed for varying levels of CAR_AUCi_ in girls and CAR_AUCg_ in boys. Region of significance tests revealed that when CAR_AUCi_ levels were ⩽−1.09 s.d. (standard deviation) and ⩽−1.71 s.d. below the mean, higher depressive symptoms significantly predicted higher levels of violence and aggression in girls, respectively. The reverse was true for the associations between depression and the two violent outcomes when CAR_AUCi_ was higher than certain levels. Specifically, when CAR_AUCi_ levels were ⩾0.65 s.d. and ⩾0.85 s.d. above the mean, higher depressive symptoms significantly predicted lower levels of girls’ violence and aggression, respectively. In addition, CAR_AUCg_ moderated the association between depressive symptoms and aggressive behaviours in boys. When the CAR_AUCg_ level was ⩽−0.24 s.d. below the mean, depressive symptoms were positively related to aggressive behaviours in boys, whereas when the CAR_AUCg_ level was ⩾3.04 s.d. above the mean, depressive symptoms were negatively associated with aggressive behaviours. The interactive effects were visualized by showing simple slopes for high in CAR (at 1 s.d. above the mean) and low in CAR (at 1 s.d. below the mean) (Fig. 1*a*–*c*). The simple slopes for girls with high and low CAR_AUCi_ are depicted in Fig. 1*a* and *b*. The simple slopes for boys with high and low CAR_AUCg_ are depicted in Fig. 1*c*.

We found that the interaction effect between depression and CAR_AUCg_ in predicting aggression in boys varied by age. The three-way interaction effect between depression, CAR_AUCg_ and age was: B (s.e.) = −0.59 (0.26), *β* = −0.10, *p* = 0.03, which suggested a stronger interaction effect when boys were older. No other age effects were found in the interaction effects.

In addition, we did sensitivity analyses to test whether the interaction effects between depression and CAR existed for subtypes of aggressive behaviours. We found that the depression × CAR_AUCg_ interaction effects occurred in predicting both proactive [B (s.e.) = −0.38 (0.12), *β* = −0.15, *p* < 0.01] and reactive physical aggression [B (s.e.) = −0.26 (0.14), *β* = −0.07, *p* = 0.06] in boys. Further, there were depression × CAR_AUCi_ interaction effects on both proactive [B (s.e.) = −0.33 (0.10), *β* = −0.20, *p* < 0.01] and reactive physical aggression [B (s.e.) = −0.23 (0.12), *β* = −0.10, *p* = 0.06] in girls.

## Discussion

In this study of 358 adolescents who were assessed annually for 3 years, we examined interactions between depressive symptoms and CAR in predicting adolescent violent outcomes using within-individual models. These models, where the effects are estimated based on variations within the same person, allowed for time-invariant factors to be accounted for, such as genetic background and early environmental experiences. We found that CAR moderated the links between depressive symptoms and adolescent violent outcomes. When CAR_AUCi_ were low, increases in depressive symptoms were positively associated with increases in violent and aggressive behaviours at the within-individual level in girls. A similar interaction pattern appeared between depressive symptoms and CAR_AUCg_ in predicting aggressive behaviours at the within-individual level in boys.

One explanation for the moderating effect of CAR is that cortisol could be associated with psychological variables, such as callous and unemotional traits, which are on the pathway between depression and violent outcomes. Low cortisol has been reported in individuals with psychopathic and callous-unemotional traits (von Polier *et al.*, 2013; Johnson *et al.*, 2014). Hence, it is possible that low cortisol may lead to disinhibition (Freitag *et al.*, 2009) and possibly more expression of callous-unemotional traits (von Polier *et al.*, 2013; Johnson *et al.*, 2014) which in turn may be linked to higher risk of violent outcomes in depressed individuals. However, this potential pathway will need to be validated. Furthermore, low CAR has been linked to conduct disorders (Pajer *et al.*, 2001), which are associated with various violent behaviours (Arseneault *et al.*, 2000). Thus, conduct disorders could potentially function as a mediator through which the risk of violent outcomes increases when an individual's depressive symptoms increased and CAR decreased. Finally, blunted CAR has been associated with poor behavioural regulation when confronting environmental stressors (Raine, 2002). Dysfunctional regulation might also lead individuals to act out violently when depressive symptoms are higher and CAR is lower. More research is required to unpack different potential mechanisms behind the interaction effects.

Our study showed differential patterns of interaction effects between CAR and depression among boys and girls. The moderating role of CAR_AUCi_ in the effects of depressive symptoms on violence and aggression was significant only in girls, whereas the moderating effects of CAR_AUCg_ appeared in predicting aggression in boys. One possibility could be that other sex hormones, such as testosterone, play a role in the interaction between depression and CAR. The participants in this study were followed from ages 15 to 17, which is an important period of sex hormonal development in adolescence with oestrogen and progesterone increasing in girls and testosterone rising in boys (Rowe *et al.*, 2004). Prior studies have showed that low cortisol predicts high levels of physical aggression particularly when the levels of testosterone are high (Popma *et al.*, 2007). It is possible that sex hormones interact with CAR and lead to differential interaction patterns between depression and CAR. For instance, it could be that depressive symptoms increase the risk of violent outcomes, but only when cortisol is low and testosterone is high. Future studies in this area should consider including sex hormones.

The negative link between depression and aggression in boys only appeared when CAR_AUCg_ was ⩾3.04 s.d. above the mean. This suggests that the protective or buffering effects of high CAR_AUCg_ on the effect of depression on aggression in boys was not as strong as that of CAR_AUCi_ on the effect in girls. That is, boys would only score low in aggression when CAR and depressive symptoms are low, whereas girls could also score low in violent outcomes when CAR and depressive symptoms are high. Further work to understand the mechanisms underlying these gender differences is required.

We found that the interaction effects between depression and CAR_AUCg_ on aggression in boys differed by age. That is, when CAR_AUCg_ was low, higher depression would lead to more aggression when the boys were older, compared with when they were younger. No age effects were found in the interaction between depression and CAR_AUCi_ in predicting violent outcomes in girls. This might be related to gender differences in development during adolescence. On average, the onset of puberty is earlier and maturation is achieved sooner in girls than boys (Colom and Lynn, 2004). It is possible that the age effect already occurred for girls, and the age range in the current study did not capture this.

Overall, the findings suggest that prevention and intervention efforts could consider the interplay between biomarkers (such as CAR) and mental health (including depressive symptoms) in predicting the risk of violence. It suggests that biological markers can provide additional information that may assist in the assessment of risk of violence and aggression, beyond current approaches that rely on historical and clinical factors.

Our study has several strengths including the longitudinal design. Repeated measures of both predictors and outcomes allowed for a within-individual design. In this study, the interaction effects referred to whether variations in CARs moderated the link between changes in depression and violent outcomes at the within-individual level. This approach enabled us to take into account time-invariant confounders such as genetic and early environmental factors (e.g. childhood adversity), which have shown consistent associations with the studied variables (Chida and Steptoe, 2009; Lewis and Plomin, 2015; Sitnick *et al.*, 2017). In addition, compared with a single measurement of CAR, our repeated measures across three annual times increased the reliability of CAR (Hellhammer *et al.*, 2007), especially given low stability of morning cortisol levels (Shirtcliff *et al.*, 2005).

However, several limitations should be noted. First, the measurement of depressive symptoms and violent outcomes was based on self-report data, which might be subject to socially desirable response bias. Presence of such bias could lead to under-reporting of violence and aggression reflected by the reliability of the violent behaviours measure (which was below 0.7 in two out of three waves). However, adolescent self-report, particularly of internalizing problems such as depression, remains an important source of information (Sourander *et al.*, 1999), and the measures we used demonstrate good external validity. Second, although we have tried to account for residual confounds using within-individual analyses and additionally controlled for alcohol drinking, substance use and stressful life events over time, other relevant time-varying confounding factors such as sex hormones might have been missed (Popma *et al.*, 2007; Mehta *et al.*, 2015; Tackett *et al.*, 2015). More studies are needed to test for bias from unmeasured time-varying confounding. Third, we noted an average decrease in CAR_AUCi_ for boys. Although this may be an artefact of a delay in sampling after awakening, negative CAR_AUCi_ could occur in accurate sampling, which has been reported (Bäumler *et al.*, 2013; Miller *et al.*, 2013; Smyth *et al.*, 2013). Furthermore, it has been shown that in general CAR_AUCi_ becomes less negative with age (Platje *et al.*, 2013). Finally, this is the first investigation, to our knowledge, of the depression–cortisol interaction effects on adolescent violent outcomes. Future research is needed to replicate these findings in different samples (e.g. clinical populations) and with different measurements of cortisol activity (e.g. hair cortisol) and reactivity (e.g. responses to social challenges) to triangulate the results.

In conclusion, this study demonstrated that CAR, including both CAR_AUCg_ and CAR_AUCi_, moderated the association between depressive symptoms and adolescent violent outcomes. The findings suggest that the CAR should be investigated further as a potential biological marker for violence in adolescents with high levels of depressive symptoms.
